# Do privacy and security regulations need a status update? Perspectives from an intergenerational survey

**DOI:** 10.1371/journal.pone.0184525

**Published:** 2017-09-19

**Authors:** Stacey Pereira, Jill Oliver Robinson, Hayley A. Peoples, Amanda M. Gutierrez, Mary A. Majumder, Amy L. McGuire, Mark A. Rothstein

**Affiliations:** 1 Center for Medical Ethics and Health Policy, Baylor College of Medicine, Houston, Texas, United States of America; 2 Institute for Bioethics, Health Policy, and Law, University of Louisville School of Medicine, Louisville, Kentucky, United States of America; University of Texas at San Antonio, UNITED STATES

## Abstract

**Background:**

The importance of health privacy protections in the era of the “Facebook Generation” has been called into question. The ease with which younger people share personal information about themselves has led to the assumption that they are less concerned than older generations about the privacy of their information, including health information. We explored whether survey respondents’ views toward health privacy suggest that efforts to strengthen privacy protections as health information is moved online are unnecessary.

**Methods:**

Using Amazon’s Mechanical Turk (MTurk), which is well-known for recruitment for survey research, we distributed a 45-item survey to individuals in the U.S. to assess their perspectives toward privacy and security of online and health information, social media behaviors, use of health and fitness devices, and demographic information.

**Results:**

1310 participants (mean age: 36 years, 50% female, 78% non-Hispanic white, 54% college graduates or higher) were categorized by generations: Millennials, Generation X, and Baby Boomers. In multivariate regression models, we found that generational cohort was an independent predictor of level of concern about privacy and security of both online and health information. Younger generations were significantly less likely to be concerned than older generations (all *P* < 0.05). Time spent online and social media use were *not* predictors of level of concern about privacy or security of online or health information (all *P* > 0.05).

**Limitations:**

This study is limited by the non-representativeness of our sample.

**Conclusions:**

Though Millennials reported lower levels of concern about privacy and security, this was not related to internet or social media behaviors, and majorities within all generations reported concern about both the privacy and security of their health information. Thus, there is no intergenerational imperative to relax privacy and security standards, and it would be advisable to take privacy and security of health information more seriously.

## Introduction

There is a commonly held assumption that members of younger generations, most notably the Millennial generation, are less concerned about the privacy and security of their information than members of older generations due to the seeming ease with which they share personal and sensitive information about themselves online via social networking and other online behaviors [1]. Attitudes toward privacy and security of personal information are especially pertinent in the era of digitization of health information, with increasing use of electronic health records (EHRs) and health information exchanges (HIEs) that facilitate health information sharing by providers, among other developments. Studies exploring public attitudes toward HIEs have shown that younger people are both less concerned about the privacy and security of their health information [2] and more likely to believe that EHRs would improve the privacy of their health information [3]. This prompts the question of the importance of strong health privacy protections in the era of the “Facebook Generation” [4]. As we continue to move health information online via HIEs, increasingly sophisticated privacy and security measures are required to protect that information from excessive disclosures and breaches. However, if younger people truly are less concerned about their health privacy and are generally more comfortable freely sharing personal and sensitive information about themselves with both friends and strangers, are efforts to strengthen health privacy protections necessary?

The increasing prevalence of online social networking certainly cannot be denied; in 2015, 65% of adults in the U.S. reported that they use social media sites compared to just 7% in 2005 [5]. The number of young adults using social media sites is even higher, with 90% of 18- to 29-year olds reporting that they use them [5]. The widespread use of these sites raises the question of whether social media users have become generally desensitized to making all manner of personal information publicly available. In fact, some go as far as to suggest that there is no longer an expectation of privacy in our current culture. In 2010, Facebook co-founder Mark Zuckerberg remarked that people have become so comfortable sharing many different types of information about themselves on the internet that privacy is no longer a “social norm” [6].

Yet, existing research actually suggests that the relationship between social media use and attitudes toward privacy and security of online information is not as straightforward as Zuckerberg claims. In a 2014 study, 80% of social media users reported that they were concerned about third parties accessing the data they shared on these sites, and 61% said they would like to increase their own efforts to protect the privacy of their online information [7].

Additionally, research has shown that American adults of all ages find their health data to be among the most sensitive of personal information, ranking it second in a list of different types of information, just after social security number, but above (though close to) the content of phone conversations, email and text messages, and physical location over time [7]. Notably, about half of respondents in a national survey reported they believed the privacy of their health information was not well protected [8].

These data suggest that people may still be concerned about their health privacy, despite the increasing prevalence of social media and a growing culture of sharing personal and sensitive information. However, the relationship between health privacy concerns, social media use, and generational cohort has not yet been directly studied. In this study, we explored perspectives toward privacy and security of health information compared to general online information between generations, and accounting for social media behaviors.

## Materials and methods

### Data collection

We developed a 45-item survey to assess participants’ perspectives toward privacy and security of two types of information: health information and general information shared online. Privacy was defined as a condition where others have limited access to the information. Security was defined as the protections that are in place to keep that information from being seen by people who do not have permission to see it. We defined health information as any information about the respondent’s physical or mental health and the healthcare they receive. Examples provided included past and current medications, illnesses, and surgeries; results from clinical tests, such as blood sugar or cholesterol level; and genetic sequencing results, such as carrier status testing results. General online information was defined as “the information you share online.” These definitions were provided in the survey before the respective sections.

We also asked about social media behaviors, use of health and fitness devices and applications, and demographic information. All items were novel measures except the Risk Propensity Scale [9]. Novel items were developed after review of relevant literature to determine study hypotheses and variable domains of interest. The study team then met to develop novel items to assess each variable. The Risk Propensity Scale, which measures an individual’s tendency to take risks, was modified into a single item from the original 7-item scale, anchored by risk avoider (1) and risk taker (9). To measure political orientation, we used a novel 15-point scale, anchored by liberal and conservative, with the midpoint labeled as moderate. We fielded a pilot survey (n = 10) in March 2016 to test survey logic and item clarity. All respondents to the pilot survey indicated that the items were clear and easy to understand, therefore we made no changes to the survey.

We conducted the survey online in the United States in March 2016 using Amazon Mechanical Turk (MTurk). MTurk is an online marketplace that is well-known for crowdsourcing recruitment for survey research [10,11]. In order to control for data quality, we restricted participation to MTurk workers with high (≥92%) approval ratings for previous MTurk tasks [12], removed incomplete surveys from analysis, restricted participants from taking the survey more than once, and used attention check questions, including an instructional manipulation question, to avoid inattentive survey takers [12]. We also restricted participation to those who were 18 years of age or older. Participants were paid US$0.50 for taking the survey. All study materials were approved by the Baylor College of Medicine Institutional Review Board (IRB).

### Data analysis

We calculated descriptive statistics including frequencies and means for participants’ characteristics and responses to survey questions. We explored differences between questions about privacy and security of online versus health information using analysis of variance (ANOVA) or paired t-tests, as appropriate. Questions about how much control they felt they had over the privacy of their online and health information and perspectives toward security of online and health information were measured on a 4-point Likert scale. Response options were dichotomized for analysis to: no control to not much control vs. a lot of control to complete control, and not at all secure to not very secure vs. somewhat secure to very secure. Linear regression was used to examine potential predictors of level of concern (continuous, scale 1–10, anchored by not at all concerned (1) and extremely concerned (10)) about the privacy and security of information shared online and health information. Potential predictors included socio-demographic and other participant characteristics, such as political and risk orientation, social media and internet behaviors, and health and fitness device use.

Participant ages were used to create generational categories, as defined by the Pew Research Center [13], Millennials (18–35 years old), Generation X (36–51 years old), and Baby Boomers (52–70 years old). As we suspected there might be differences within the Millennial generational category, we conducted an exploratory analysis and created two Millennial categories: Younger Millennials, 18–27 years (47.7% of Millennials), and Older Millennials, 28–35 years (52.3% of Millennials). We then explored whether there were differences within the Millennial category in level of concern (continuous, scale 1–10, anchored by not at all concerned (1) and extremely concerned (10)) about the privacy and security of health information using ANOVA. Given the data distribution, response options were dichotomized for analysis to not concerned (1–5) and concerned (6–10). Finally, we assessed differences in levels of concern about privacy and security of health information between the new expanded generational categories using ANOVA. Statistical analyses were conducted using SPSS 23 (IBM Corp., Armonk, N.Y., USA). All *P* values were two-sided and with statistical significance set at *P* < 0.05.

## Results

In total, 1443 people provided at least some answers to the survey. We excluded responses from 61 people who did not complete the survey, from 63 people who answered one or more attention check questions incorrectly, and from 9 people who were members of the Silent Generation (71 years and older) due to small group size (n = 9), leaving 1310 (90.8%) respondents for analysis.

Participants’ sociodemographic characteristics are presented in Table 1. Respondents were 50% male, 55% had an annual household income of less than $49,000, and the majority were non-Hispanic white (78%). They had a mean age of 36.3 years (SD = 12.4); 59% were 18–35 (Millennial Generation), 26% were 36–51 (Generation X), 14% were 52–70 (Baby Boomers). Fifty-four percent were college graduates or higher and participants leaned slightly liberal.

**Table 1:** Participant characteristics.

Characteristic–N (%) unless otherwise noted		n = 1310
*Age*		
	Mean in years (SD)	36.1 (12)
	Median in years	33
Generational group*		
	Millennials (born after 1980, <36y.o.)	772 (59)
	Generation X (born 1965–1980, 51-36y.o.)	348 (26)
	Baby Boomers (born 1946–1964, 70-52y.o.)	190 (15)
*Gender*^†^		
	Male	658 (50)
*Race/Ethnicity*		
	Hispanic or Latino	101 (8)
	Non-Hispanic White	1017 (78)
	African American	73 (6)
	Asian	90 (7)
	Non-Hispanic Other^‡^	29 (2)
*Education*		
	High school graduate/GED or less	153 (12)
	Some college or post-high school training	448 (34)
	College graduate or higher	709 (54)
*Annual household income*		
	≤ $49,000	712 (54)
	$50,000 - $99,999	440 (34)
	≥ $100,000	158 (12)
*Health insurance source*		
	Employer	478 (37)
	Parents or partner	280 (21)
	Healthcare Marketplace	111 (8)
	Medicaid or state insurance	142 (11)
	Medicare	57 (4)
	Private insurance	61 (5)
	Do not have health insurance	156 (12)
	Other	25 (2)
*Political orientation*		
	Mean continuous scale, Liberal (-7)—Moderate (0)— Conservative (7)	-1.5 (4.2)
*Risk orientation*		
	Mean continuous scale, Risk avoider (0)—Risk tasker (10)	4.1 (2.0)

*Generation groups defined by Pew Research Center [13].

^†^Categories do not sum to 1310 because of participant non-response.

^‡^Non-Hispanic Other includes: American Indian or Alaskan Native (13); Native Hawaiian or Other Pacific Islander (4), and Other (12).

The majority of participants (79%) indicated that they spend four or more hours online per day. Ninety-four percent reported that they use social media sites, 80% checked social media sites more than once per week, and 50% posted to social media sites more than once per week. Thirty-five percent reported that they use health and fitness devices or applications (such as FitBit, Nike+, Apple’s Health Kit, etc.).

The majority of respondents felt that they had little to no control over the privacy of the information they shared online (63%), and that information was not at all to not very secure (60%). They were also concerned about both the privacy (69%) and the security (75%) of their online information. When sharing data online, respondents were most concerned about: identify theft (76%), embarrassment (46%), and financial loss (42%).

**Fig 1:**
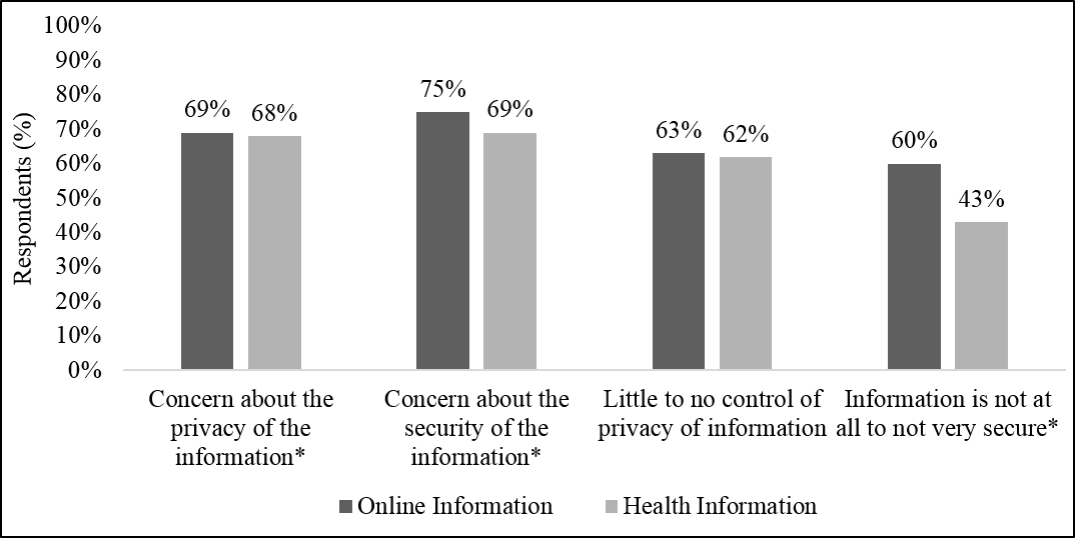
Privacy & security of general online information vs. health information. Concern about privacy and security of information was measured on a 1–10 scale. Percentages represent respondents who selected 6–10, indicating concern. Control of privacy of information was measured on a Likert scale with response options of No control, Not much control, A lot of control, and Complete control. Percentages reflect respondents who selected A lot to Complete control. Security of information was measured on a Likert scale with response options of Not at all secure, Not very secure, Somewhat secure, and Very secure. Percentages reflect respondents who selected Somewhat to Very secure. *Significant at P < 0.05.

Similarly, a majority of respondents reported being concerned about the privacy and security of their health information (68% and 69%, respectively; Fig 1). While there was no difference in respondents’ feelings about the lack of control they had over the privacy of their health information compared to their online information (62% vs. 63%, respectively), significantly more respondents felt that their online information was not secure compared to their health information (60% vs. 43%), though confidence in the security of both types of information was relatively low (*P* < 0.001).

In multivariate regression models controlling for demographic characteristics, political and risk orientations, social media and internet behaviors, and health and fitness device and application use, we found that generational cohort was an independent predictor of level of concern about privacy and security of both online and health information. Younger generations were significantly less likely to be concerned about the privacy and security of their online and health information than older generations (all *P* < 0.05) (Tables 2 and 3). Those reporting higher education levels were more concerned about the privacy of their online and health information and the security of their health information (all *P* < 0.05). Compared to respondents who identified as risk takers, risk avoiders were more concerned about the privacy and security of their online (but not health) information (each *P* < 0.01). Gender was an independent predictor of level of concern about security of online information (female participants were more concerned than male participants), and race and ethnicity was an independent predictor of level of concern about privacy of health information (*P* < 0.05) (minority racial and ethnic groups were significantly more concerned about the privacy of their health information than non-Hispanic whites). Contrary to our expectation, we found that the amount of time respondents spent online, frequency of checking social media, frequency of posting on social media, and use of health and fitness devices and applications were *not* predictors of level of concern about privacy or security of online or health information (all *P* > 0.05).

**Table 2:** Predictors of level of concern about privacy of online and health information*.

	*Online Information*		*Health Information*	
Characteristic/Online habits	*Β coefficient*	*95% Confidence interval*	*Β coefficient*	*95% Confidence interval*
Education level	0.224	0.021–0.427^†^	0.268	0.061–0.475^†^
Generation group	0.211	0.022–0.400^†^	0.411	0.218–0.604^†^
Gender	0.238	-0.035–0.510	0.252	-0.027–0.531
Income level	-0.130	-0.332–0.072	-0.102	-0.309–0.104
Race/Ethnicity	0.122	-0.103–0.346	0.267	0.038–0.497^†^
Political orientation	0.012	-0.021–0.044	0.015	-0.018–0.048
Risk orientation	-0.098	-0.168 - -0.028^†^	-0.033	-0.104–0.038
Frequency online	-0.056	-0.170–0.058	-0.072	-0.189–0.044
Frequency check social media	-0.113	-0.309–0.082	-0.041	-0.241–0.159
Frequency post to social media	-0.038	-0.232–0.156	0.190	-0.009–0.388
Health & fitness device/app users	0.134	-0.149–0.417	0.035	-0.254–0.325

*Level of concern measured on continuous scale, anchored by 1 = Not at all concerned to 10 = Extremely concerned.

^†^Indicates *P* value < 0.05.

**Table 3:** Predictors of level of concern about security of online and health information*.

	*Online Information*		*Health Information*	
Characteristic/Online habits	*Β coefficient*	*95% Confidence interval*	*Β coefficient*	*95% Confidence interval*
Education level	0.128	-0.072–0.327	0.211	0.000–0.421 ^†^
Generation group	0.315	0.129–0.500^†^	0.377	0.181–0.574 ^†^
Gender	0.322	0.053–0.591^†^	0.156	-0.128–0.440
Income level	-0.088	-0.287–0.110	-0.149	-0.359–0.061
Political orientation	0.007	-0.025–0.039	0.032	-0.002–0.065
Race/Ethnicity	0.103	-0.118–0.324	0.141	-0.093–0.375
Risk orientation	-0.090	-0.159 - -0.022^†^	-0.032	-0.104–0.041
Frequency online	-0.066	-0.178–0.046	-0.047	-0.165–0.072
Frequency check social media	-0.017	-0.209–0.176	0.024	-0.180–0.227
Frequency post to social media	-0.108	-0.299–0.083	0.057	-0.145–0.258
Health & fitness device/app users	-0.013	-0.292–0.265	-0.043	-0.251–0.338

*Level of concern measured on continuous scale, anchored by 1 = Not at all concerned to 10 = Extremely concerned.

^†^Indicates *P* value < 0.05.

While Millennials were less concerned about the privacy and security of their health information, more Millennials (43%) than respondents of Generation X (30%) and the Baby Boomer Generation (32%) reported feeling that they had control over the privacy of their health information. Additionally, more Millennials (61%) than Generation X (55%) and Baby Boomers (48%) reported that their health information was secure.

When asked how their level of concern has changed over the past 10 years, the majority of respondents in all generational cohorts reported that they are more concerned now about the privacy (54% of Millennials, 66% of Generation X, 65% of Baby Boomers) and security of their health information (53% of Millennials, 62% of Generation X, 63% of Baby Boomers).

**Fig 2:**
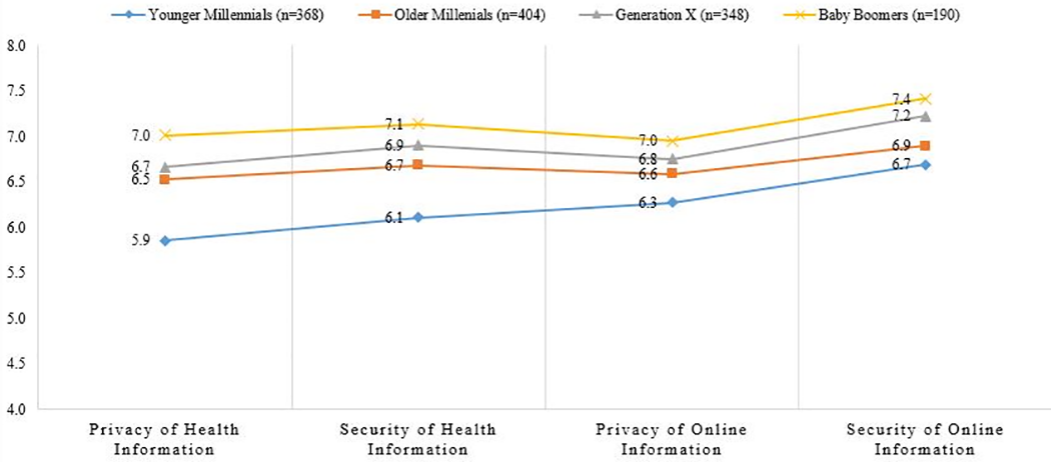
Concern about privacy & security of general online vs. health information. Younger Millennials 18–27 years old, Older Millennials 28–35 years old, Generation X 36–51 years old, Baby Boomers 52–70 years old. Concern about privacy and security of information was measured on a 1–10 scale, anchored by 1 = Not at all concerned to 10 = Extremely concerned.

We also analyzed level of concern about privacy and security of health information within the Millennial Generation using ANOVA. We found that when we split those respondents into an Older Millennial group (ages 28–35 years) and a Younger Millennial group (18–27 years), Younger Millennials were less concerned about both the privacy [*F*(3, 1309) = 11.969, *p* = .000] and the security [*F*(3, 1309) = 9.743, *p* = .000] of health information than all other generations, and Older Millennials responded more similarly to the Generation X and Baby Boomer respondents (Fig 2).

## Discussion

As noted above, it is widely assumed that members of younger generations are less concerned about their privacy than members of older generations because they have become desensitized to sharing personal information online via social media and other online activities. We have previously argued [4] that there is a risk this assumption could invite unwarranted conjecture that rigorous efforts to protect the privacy of health information are no longer necessary, and that the cost of protecting privacy in the electronic era of healthcare outweighs the need.

We found that a majority of all respondents, including Millennials, were concerned about the privacy and security of their health information. Millennials, however, were somewhat less concerned about the privacy and security of their health information than respondents of older generations. In fact, when looking at variation within the Millennial cohort, we found that younger Millennials (18–27 years) were less concerned about the privacy and security of their health information than older Millennials (28–35 years), whose views more closely matched those of the older generations. Notably, however, this lower level of concern was not associated with social media use, other online behavior, or use of health and fitness devices and applications. The reason for these lower levels of concern, therefore, is not a straightforward effect of current social media and internet behavior.

It is possible that Millennials were less concerned about the privacy and security of their health information simply because they are likelier than members of older generations to be in good health with less significant and fewer health issues overall, thus lessening the concern about that information being disclosed or breached. As such, the difference in views, particularly the difference between the older and younger Millennials, may be due to differences in life stage rather than an ideological difference between the generations. As Millennials age, their health status along with their views may change. It is important to note here that current health privacy protections for young adults are also protections for future middle-aged adults.

Millennials may also have been less concerned because they felt they had more control over the privacy of their health information and that their health information was secure (even if this confidence is potentially overly optimistic, or perhaps reflective of a level of overconfidence due to inexperience). Additionally, respondents reported similar levels of concern about the privacy and security of their health information and their online information. The majority of respondents also reported that they are more concerned about the privacy and the security of their health information now compared to 10 years ago (though it is uncertain how these questions performed for the Millennials aged 18–27 who would have reflected back to ages 8–17). These findings suggest that concern about privacy and security of health information may be linked to the onset of the electronic era of healthcare, in which health data are opened up to a host of new digital-based threats, including ransomware attacks [14]. Thus, allowing rigorous standards of health security to weaken as a casualty of the move of healthcare into the electronic age would be unjustified.

Our findings must be considered within the limitations of the study. First, MTurk samples, though more diverse than many convenience samples [10], are not representative of the sociodemographic characteristics of the population of the U.S. and have been shown to be slightly younger and more educated [15]. Compared to national statistics collected on adults by the U.S. Census Bureau, our survey respondents were younger (the median age of our participants was 33 years vs. 37 years nationally) [16], mostly non-Hispanic white (78% of our participants reported being non-Hispanic white vs. 64% non-Hispanic white nationally) [17], and were more educated (54% of our participants held at least a Bachelor’s degree vs. 33% of adults 25 and older nationally) [18]. The amount of time our participants reported spending online is similar to available national data, with 73% of Americans reporting going online at least daily, and 63% going online several times a day or more in 2015 [19], but more of our participants were social media users, 94% of our participants compared to 77% of adults in a national survey in 2016 [20]. Our respondents’ use of health and fitness devices and applications was similar to 2016 national data showing 33% of Americans used a health application, and 21% used a wearable device to manage their health [21].

Second, while restricting the survey to MTurk users with a high approval rating improved the integrity of our results [12], the legitimacy of MTurk survey respondents’ responses cannot be completely controlled. Third, those using the MTurk platform are likely more comfortable sharing information online, though some research has shown that MTurk users have more privacy concerns than the general public [22]. Therefore, these data may not reflect the privacy and security concerns of the general US population. Nevertheless, this study is the first to challenge the notion that younger people are less concerned or even unconcerned about the privacy of their health information because of their willingness to share information online. Future research should explore attitudes toward health privacy and security with a larger and more representative sample. Finally, we are unable to distinguish between effects of generational cohort vs. age cohort as described previously. Thus, while it appears that there is an ideological shift within the Millennial generation, we are unable to determine whether there are truly two Millennial subgroups, older and younger, or whether the difference in concerns about privacy and security of health information is simply due to the difference between more and less mature adults. Future research should follow these perspectives over time to determine whether this split holds as the Millennials mature.

Although our data support the claim that Millennials have lower levels of concern about privacy than other generations, this is not directly related to their internet or social media behaviors, and majorities within all generations report concern about both the privacy and the security of their health information. Thus, there is no intergenerational imperative to relax health privacy and security standards under the Health Insurance Portability and Accountability Act and other laws that protect health-related information. In fact, since respondents of all generations report being more worried now than they were 10 years ago, and in light of recent hacking events targeting health data, as well as new efforts to collect and widely share health information for research purposes, it would be advisable to take privacy and security of health information even more seriously.

## Supporting information

S1 File Health privacy security dataset. (XLSX)

S2 File Complete MTurk survey. (DOCX)
