# Supplementary material for: Do privacy and security regulations need a status update? Perspectives from an intergenerational survey
Source: PLoS One. 2017 Sep 19;12(9):e0184525. doi: 10.1371/journal.pone.0184525 (PMC5604938; doi:10.1371/journal.pone.0184525)
Supplement: S2 File — (DOCX) [file pone.0184525.s002.docx]

Do privacy and security regulations need a status update? Perspectives from an intergenerational survey

S. Pereira et al.

SURVEY:

We are inviting you to participate in a research study being conducted by Amy McGuire, JD, PhD and Mary Majumder, JD, PhD, in the Center for Medical Ethics and Health Policy at Baylor College of Medicine, and Mark Rothstein, JD in the Institute for Bioethics, Health Policy, and Law at the University of Louisville School of Medicine.

Participation in this study includes filling out an online survey about how you use social media, your views toward privacy and security of your information, and how you feel about different groups having access to your health information. We will also ask you some questions about yourself, including your age, race and ethnicity, education, and income. We will NOT ask you for any personally identifying information (like your name or email address).

This survey should take you about 15 minutes to complete. Your participation is completely voluntary, and you may stop at any time for any reason.

If you are willing to participate, please click the “Next” button below.

In this first section of the survey, we will ask you questions about your social media use, the types of information you share online, and what you think about the privacy and security of your information.

1. **On average, how often are you online each day?**

- 0-1 hours a day
- 1-3 hours a day
- 4-6 hours a day
- 7-9 hours a day
- 10-12 hours a day
- 13+ hours a day

1. **Do you use any social media sites? For example: Facebook, Twitter, Instagram, etc.**

- Yes
- No

1. **On average, how often do you post to social media sites?**

- I never post to social media
- A few times a year
- A few times a month
- A few times a week
- Almost every day
- Once a day
- 2-5 times a day
- 6-10 times a day
- More than 10 times a day

1. **On average, how often do you check or view social media sites?**

- A few times a year
- A few times a month
- A few times a week
- Almost every day
- Once a day
- 2-5 times a day
- 6-10 times a day
- More than 10 times a day

Privacy of your information refers to a condition where others have limited access to information about you. Some ways in which privacy of your information may be protected are by YOU controlling the following:

- what information is collected about you
- who it is collected by
- who it is shared with
- what purposes it is shared for

An example of a way someone might feel his privacy was violated would be companies collecting and sharing information about him from his social media account without him having control over that collection and sharing.

1. **How much control do you feel you have over the privacy of the information you share online?**

- No control
- Not much control
- A lot of control
- Complete control

Security of information refers to the protections that are in place to keep your information from being seen by people who do not have permission to see it. An example of how online information is kept secure is the use of encryption.

An example of a security breach would be if a third party hacked into a social media website’s database and stole users’ personal information.

1. **How secure do you think the information you share online is?**

- Not at all secure
- Not very secure
- Somewhat secure
- Very secure

1. **How concerned are you about:**

|  | **Not at all Concerned**  **1** | **2** | **3** | **4** | **5** | **6** | **7** | **8** | **9** | **Extremely Concerned**  **10** |
| --- | --- | --- | --- | --- | --- | --- | --- | --- | --- | --- |
| The privacy of the information you share online |  |  |  |  |  |  |  |  |  |  |
| The security of the information you share online |  |  |  |  |  |  |  |  |  |  |

1. **Are you concerned about any of the following harms associated with the privacy and/or security of the information you share online?**
   - Identity theft
   - Embarrassment
   - Loss of education opportunities
   - Loss of employment opportunities
   - Financial loss
   - Discrimination
   - I am not concerned about any harms
   - Other, please specify: [free text box]
2. **If you chose more than one harm in the previous question, which of the following harms are you most concerned about?**
   - Identity theft
   - Embarrassment
   - Loss of education opportunities
   - Loss of employment opportunities
   - Financial loss
   - Discrimination
   - Other, please specify: [free text box]
3. **Do you use any health and fitness devices and/or smartphone applications? For example: Fitbit, Garmin, Nike+, Apple's Health Kit, etc.**

- Yes
- No

The next section focuses on your health information and how private and secure you think your health information is.

“Health information” means any information that is about your physical or mental health and the healthcare you receive.

Examples of health information include:

- past and current medications, illnesses, and surgeries
- results from clinical tests, such as blood sugar or cholesterol level
- genetic sequencing results, such as carrier status testing results

Privacy of your information refers to a condition where others have limited access to information about you. Some ways in which privacy of your health information may be protected are by YOU controlling the following:

- what information is collected about your health
- who it is collected by
- who it is shared with
- what purposes it is shared for

An example of a way someone might feel the privacy of his health information was violated would be a pharmacy sharing information about his prescriptions with a pharmaceutical company for marketing purposes without his knowledge.

1. **How much control do you feel you have over the privacy of your health information?**

- No control
- Not much control
- A lot of control
- Complete control

Security of health information refers to the protections that are in place to keep your health information from being seen by people who do not have permission to see it. An example of how health information is kept secure is the use of security software on computers. An example of how the security of your health information could be breached would be if a third party hacked into your healthcare provider’s computer system and stole your health information.

1. **How secure do you think your health information is?**

- Not at all secure
- Not very secure
- Somewhat secure
- Very secure

1. **How concerned are you about:**

|  | **Not at all Concerned**  **1** | **2** | **3** | **4** | **5** | **6** | **7** | **8** | **9** | **Extremely Concerned**  **10** |
| --- | --- | --- | --- | --- | --- | --- | --- | --- | --- | --- |
| The privacy of your health information |  |  |  |  |  |  |  |  |  |  |
| The security of your health information |  |  |  |  |  |  |  |  |  |  |

1. **Has your level of concern about the privacy of your health information changed over the last 10 years?**
   - Yes, I am more concerned now
   - Yes, I am less concerned now
   - No, I have the same level of concern
2. **Has your level of concern about the security of your health information changed over the last 10 years?**
   - Yes, I am more concerned now
   - Yes, I am less concerned now
   - No, I have the same level of concern

In this last section, we will ask you some basic questions about yourself.

1. **What is your age?**
2. **How do you describe your gender? Please select all that apply.**
   - Male
   - Female
   - Do not identify as male or female
   - Different identity (please state): [text box]
3. **What is the highest level of education you completed?**
   - None
   - Grade school (grades 1-8)
   - Some high school (grades 9-12)
   - High school graduate or GED
   - Post high school training other than college or associate’s degree (for example: vocational or technical program)
   - Some college but no degree
   - Bachelor’s degree (for example: BA, AB, BS)
   - Master’s degree (for example: MA, MS, MEng, Med, MSW, MBA)
   - Doctorate or professional degree (for example: MD, JD, PhD, EdD)
4. **What is your current employment status? Please select all that apply.**
   - Employed full-time (more than or equal to 32 hours per week)
   - Employed part-time (less than 32 hours per week)
   - Full-time student
   - Part-time student
   - Unemployed, seeking work
   - Homemaker
   - Unable to work due to disability
   - Retired
5. **What was your total household income (before taxes) from all sources in the last year?**
   - Less than $10,000
   - $10,000 to $19,999
   - $20,000 to $34,999
   - $35,000 to $49,999
   - $50,000 to $74,999
   - $75,000 to $99,999
   - $100,000 to $149,999
   - $150,000 or more
6. **Are you of Hispanic, Latino, or Spanish origin?**

- No
- Yes

1. **How do you describe your race? Please select all that apply.**
   - American Indian or Alaska Native
   - Asian
   - Black or African American
   - Native Hawaiian or Other Pacific Islander
   - White
   - Other, please specify: [free text box]
2. **In what U.S. state or U.S. territory do you currently live?**

[Dropdown list with all states & territories]

1. **Where do you obtain your health insurance?**
   - Through my employer
   - Through my parents or my partner
   - I purchase my healthcare insurance through the HealthCare Marketplace (US federal government insurance plans)
   - I have Medicaid or other state insurance
   - I have Medicare
   - I purchase my own private health insurance (outside of employer)
   - I do not have health insurance
   - Other, please specify: [free text box]
2. **Please choose the spot on the scale that best indicates your political orientation.**

| **Liberal** |  |  |  |  |  |  | **Moderate** |  |  |  |  |  |  | **Conservative** |
| --- | --- | --- | --- | --- | --- | --- | --- | --- | --- | --- | --- | --- | --- | --- |
|  |  |  |  |  |  |  |  |  |  |  |  |  |  |  |

1. **I view myself as a:**

| **Risk Avoider**  **1** | **2** | **3** | **4** | **5** | **6** | **7** | **8** | **9** | **Risk Taker**  **10** |
| --- | --- | --- | --- | --- | --- | --- | --- | --- | --- |
|  |  |  |  |  |  |  |  |  |  |
